# Evaluation of the usefulness of determining the level of selected inflammatory biomarkers and resistin concentration in perivascular adipose tissue and plasma for predicting postoperative atrial fibrillation in patients who underwent myocardial revascularisation

**DOI:** 10.1186/s12944-022-01769-w

**Published:** 2023-01-09

**Authors:** M. Rachwalik, M. Matusiewicz, M. Jasiński, M. Hurkacz

**Affiliations:** 1 Clinical Department of Cardiac Surgery, Department of Cardiac Surgery and Heart Transplantation, Institute of Heart Diseases, Wroclaw Medical University, Wroclaw, Poland; 2 Department of Biochemistry and Immunochemistry, Wroclaw Medical University, Wrocław, Poland; 3 Childrens Memorial Health Institute in Warsaw, Warsaw, Poland; 4 Department of Clinical Pharmacology, Wroclaw Medical University, Borowska 211 Str 50-556, Wrocław, Poland

**Keywords:** Postoperative atrial fibrillation, Resistin, Coronary artery bypass graft, Perivascular adipose tissue

## Abstract

**Background:**

The development of coronary artery disease (CAD) is related to the impaired quantity and composition of inflammatory proteins found in plasma and tissue, such as interleukin 6 (IL-6), adipokines, and resistin. Therefore, the level of plasma resistin in patients with advanced CAD could be indicative of the condition of epicardial adipose tissue and thus have an impact on the frequency and severity of postoperative complications in the form of paroxysmal atrial fibrillation.

**Methods:**

The study included 108 patients who qualified for elective coronary artery bypass grafting (CABG) surgery from 2017 to 2020 and were categorized into two groups. The first group consisted of patients who developed atrial fibrillation in the postoperative period – the AF group, and the second group included patients who did not have arrhythmia – the non-AF group. The analysis incorporates the history, course of treatment, anthropometric characteristics of the test subjects, biochemical laboratory tests, and echocardiography. Perivascular adipose tissue (PVAT) sections were surgically harvested from the area of the left coronary trunk.

**Results:**

The resistin levels in the PVAT were significantly higher in the AF group than in the non-AF group (*P* = 0.000015). Similarly, plasma resistin levels increased significantly in the AF group compared to the non-AF group (*P* = 0.044). The values of other analyzed variables were not significantly different (analysis performed using the Mann–Whitney U test). Spearman’s rank-order correlation technique found a correlation between resistin in PVAT and plasma (r = 0.5933; *P* < 0.0001) in the whole study group, as well as in the AF group (r = 0.4782; *P* = 0.021) and the non-AF group (r = 0.4938; *P* < 0.0001). A correlation arose between the level of resistin in PVAT and the level of hsCRP (r = 0.3463; *P* = 0.005) in the whole study group and the non-AF group (r = 0.4448; *P* = 0.0011); however, no such correlation appeared in the AF group (r = 0.3076; *P* = 0.306).

**Conclusions:**

Elevated levels of plasma resistin, which reflect PVAT resistin levels in patients qualified for myocardial revascularisation, may be associated with postoperative atrial fibrillation complications.

**Supplementary Information:**

The online version contains supplementary material available at 10.1186/s12944-022-01769-w.

## Background

Coronary artery disease (CAD), one of the most common civilization disorders, is among the most significant health challenges of the twenty-first century [1]. Despite advancements in various treatment guidelines, heart disease continues to be the leading cause of death worldwide [2]. Therefore, the search continues for factors that could enable the prediction and prevention of disease development [3].

Atrial fibrillation (AF) often affects patients with CAD, while almost 4% of patients in the general population have reported suffering from AF [4]. AF is a major risk factor for ischemic stroke; it causes significant morbidity and mortality and increases the economic healthcare burden [5]. This is particularly true for atrial fibrillation after myocardial revascularisation surgeries such as coronary artery bypass grafting (CABG). Postoperative atrial fibrillation (AF) is often the cause of prolonged patient hospitalization. Atrial fibrillation is responsible for a 2-fold increase in 30-day all-cause mortality and 6-month postoperative mortality [6]. AF is also associated with an increased risk of stroke, bleeding, infection, and renal failure [7]. The long-term consequences remain unknown [8]. De novo postoperative atrial fibrillation may occur in up to 20–30% of cardiac surgery patients [9].

The causes of postoperative atrial fibrillation are multifactorial; some are associated with comorbidities, while others are related to surgical injuries. The origin of postoperative AF is the subject of ongoing research. The latest findings suggest that the development of AF is related to proinflammatory factors, e.g., IL-6 [10, 11]. Perivascular adipose tissue (PVAT) plays an essential role in the development of AF [12]. PVAT is a reservoir of lipids that occur physiologically near coronary arteries on the surface of the myocardium. It is a source of energy for cardiomyocytes and insulates the autonomic ganglia and nerve fibers. PVAT has paracrine properties and acts by releasing oxidative and inflammatory stress modulators. The inflammatory response is believed to cause local damage to the cardiac conduction system [13].

Adipocytokines secreted by PVAT, present in peripheral blood, may activate nonspecific inflammation. These proteins, discovered at the end of the twentieth century, are components of adipose tissue and have a significant impact on the development of atherosclerosis, insulin resistance, and cancer, as well as on the course of inflammation. Resistin, one of the representatives of this group of proteins, has been identified as an essential marker of atherosclerosis development. Analytical methods of peripheral blood and adipose tissue, such as PVAT, may be used to determine resistin levels [12, 14].

During perioperative stress, adipokines may be an indirect reason for the occurrence of AF [15]. Resistin does not appear to have direct arrhythmogenic effects. The presence of resistin in adipocytes may mediate their paracrine effects and cause local inflammation in myocardial cells by activating proinflammatory and inflammatory cytokines. This subsequently causes necrosis or dysfunction of the cell membrane. In PVAT, adipose tissue is in contact with epicardial coronary arteries. Thus, chronic local inflammation may influence the abnormal development of fibrous tissue and infiltration of conducting tissue [16].

This is the first study to assess whether measuring plasma adipokine levels, such as resistin, reflects their level in PVAT and, therefore, predicts postoperative complications.

## Materials and Methods

### The aim

The study aims to assess the clinical value of the perioperative determination of the level of epicardial resistin in patients with coronary artery disease and the likelihood of AF based on preoperative measurement of plasma resistin levels and other indicators, such as IL-6, hsCRP, troponin, and BMI (body mass index).

### Study description

Characteristics of the participants: The study analyses a prospective study performed on a cohort of 108 patients referred for elective coronary artery bypass grafting (CABG) due to advanced CAD by the Clinical Department of Cardiac Surgery of the University Clinic Hospital in Wroclaw who underwent cardiac surgery treatment – myocardial revascularisation with extracorporeal circulation. The patient’s average length of hospital stay was 6.5 days (SD 3 days). Their baseline clinical and echocardiography data were recorded. The patients included in the study were randomly selected from all patients (*n* = 2276) undergoing cardiac surgery at the Clinical Department in the period covered by this study, i.e., from February 2017 to October 2020. The following criteria had to be met:

The inclusion/exclusion criteria were as follows: triple-vessel coronary artery disease (3VD), presence of sinus rhythm on the electrocardiogram (ECG), age < 80 years old, receiving statin treatment for four weeks or longer in the period before the cardiac surgical intervention, and written consent to participate in the study. Triple-vessel disease (3VD) corresponds to advanced atherosclerosis in which coronary lesions can result from hypercholesterolemia and the effects of factors in epicardial tissue. In these patients, we hypothesized a higher resistin content and better quality of homogenates using epicardial tissue collection marked in the diagram relative to patients with less advanced coronary artery disease. The study included selected patients who were admitted to the hospital and already receiving cardiological treatment for CAD. In addition to drugs for hypertension, B-blockers, and aspirin, most were taking statins. We included patients taking similar drug treatments in the study to standardize patients.

Among the inclusion criteria, patients with a positive history of AF were not included in the study. Patients included in the study were not previously treated with antiarrhythmic drugs.

The exclusion criteria were severe or moderate valvular heart disease that required additional surgery and left ventricular ejection fraction (LVEF) lower than 30%, insulin-dependent diabetes (diabetes diagnosis based on the guidelines of the Polish Diabetology Society), end-stage renal disease, rheumatic diseases, infections, or cancer.

From initial diagnosis to surgery, pharmacotherapy with hypolipidemic drugs (rosuvastatin) was introduced and continued for at least four to five weeks. Each patient received a 12-lead ECG before surgery. Postoperative AF was diagnosed after electrocardiographic confirmation of arrhythmia in each patient. ECGs of all patients were monitored 24 hours per day in the intensive care unit and later in the cardiac surgery unit for full three days postoperatively. During the following four days, resting ECGs were performed daily according to ward routine and whenever the patient reported abnormal rhythms or malaise. A conventional 12-lead ECG was performed when AF was recorded on the monitor or when a medical assessment heightened suspicions of arrhythmia. AF was defined according to the Guidelines for the management of atrial fibrillation of the European Society of Cardiology published in 2016.

The diagnosis of AF required documentation of the rhythm with an electrocardiogram showing AF. Electrocardiographic features of AF included irregular R-R intervals (when atrioventricular conduction was not impaired), absence of distinct repetitive P-waves, and irregular atrial activations. By convention, an episode lasting at least 30 seconds was diagnostic for clinical AF in the study group.

Pharmacotherapy: Patients underwent routine pharmacotherapy consisting of beta-blockers, ACE inhibitors, statins (each patient received rosuvastatin), nitrates, and subcutaneous low-molecular-weight heparin (LMWH). Patients undergoing CABG surgery were not taking oral sodium-dependent glucose transporter 2 inhibitor (SGLT2) antidiabetic drugs during the described follow-up or before surgery. Drugs were administered based on pharmacotherapy guidelines and adjusted to the patient’s weight and clinical condition.

### Data collection

The material for biochemical analyses was collected from October 2017 to December 2020. The Bioethics Committee approved the study (No. KB-392/2016), and each patient signed informed consent to participate. All patients were admitted to the department and underwent elective surgery. Patients were divided into two groups depending on the occurrence of atrial fibrillation in the postoperative period. The first group consisted of patients who developed AF – the AF group (*N* = 23), while the second group included patients without arrhythmia – the non-AF group (*N* = 84).

The study was performed with the support of a local university, grant number: ST. C050.21.033.

### Surgical procedures

Standard sternotomy was utilized during CABG surgeries. Cardiopulmonary bypass (CBP) was performed using an aortic cannula inserted into the ascending aorta and a venous return cannula inserted into the right atrial appendage. The average duration of aortic cross-clamping was 40–45 min. The duration of aortic cross-clamping during bypass surgery and the postoperative troponin levels in patients were compared in the study groups. There were no significant differences between the two groups. After cardiac arrest, PVAT around the left main coronary artery (LMCA) was sampled using surgical scissors and a blade. A small portion of the sample (3 × 3 × 3 mm) was immediately frozen at − 80 °C and stored for further analysis. The samples of PVAT collected intraoperatively had a mean weight of 0.0303 g (SD - 0.0166 g). For patient safety, fragments of the left atrium (LA) were not sampled for the study, as resistin levels in PVAT and LA should be the same. The left artery (LA) remains intact during a typical CABG surgery PVAT tissue was obtained from the LMCA region, which is anatomically positioned next to the LA.

**Fig. 1:**
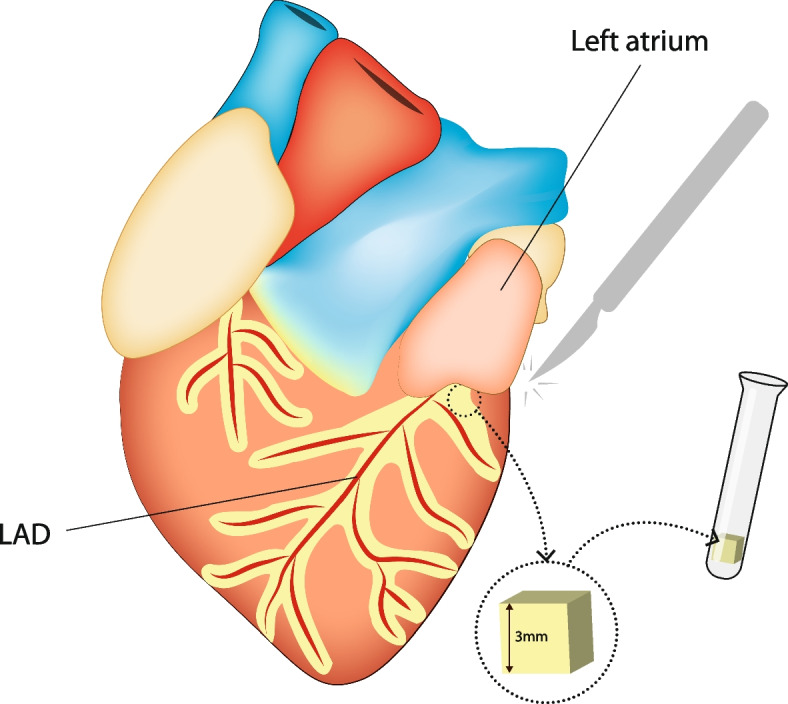
The method of PVAT collection from the area around the LMCA during cardiac surgery

Following sampling, myocardial revascularisation continued in a typical manner. Figure 1 shows a diagram of the method of PVAT collection from the area around the LMCA during cardiac surgery for biochemical analyses.

### Sample collection

Venous blood samples were also collected from patients in the hospital before CABG to determine plasma levels of selected biomarkers, such as resistin and IL-6. The samples were centrifuged in a laboratory centrifuge at 5000 rpm for 10 minutes; the obtained plasma was frozen to − 80 °C. Other biochemical parameters, such as the hsCRP level, total cholesterol (Chol), HDL fraction, LDL fraction, triglycerides (TG), glucose, bilirubin, creatinine, the activity of AspAT and ALAT enzymes, as well as a full blood count, were determined before and after the surgery by the Hospital’s Diagnostic Laboratory with the application of standard diagnostic methods using the biochemical analysers Olympus AU480 (Beckman Coulter), Cobas Integra 400 plus by Roche, photometry, turbidimetry and potentiometric methods. A FastPrep homogenizer (MP Biomedicals, Santa Ana, CA, USA) was used to homogenize the PVAT samples, and then PBS buffer containing PMSF was used. Centrifugation was performed twice for 10 min at 14,000 *xg* and 4 °C. The second centrifugation was performed just before the analysis. PVAT resistin levels and plasma resistin levels were measured using the Human Resistin Quantikine ELISA kit (R&D Systems, Minneapolis, MN, USA) according to the manufacturer’s instructions and were expressed as ng/g of tissue or ng/mL of plasma. IL-6 levels were measured using the Human IL-6 Quantikine ELISA kit (R&D Systems).

### Statistical analyses

The analysis of quantitative variables (i.e., those expressed by number) was performed by calculating the mean, standard deviation, median, quartiles (Q1 and Q3), and IQR (interquartile range). The normality of the distribution of the variables was verified using the Shapiro–Wilk test and histograms. The results indicated an abnormality in the distribution of the majority of the assessed variables; therefore, nonparametric tests were used for further statistical tests. The quantitative variables were compared for the two groups using the Mann–Whitney U test. The correlation between quantitative variables was analyzed using Spearman’s rank correlation coefficient. A significance level of 0.05 was adopted in the analysis; therefore, all *P* values ​​below 0.05 indicated significant correlations. An analysis of the power of tests for independent samples was performed to assess the predictive usefulness of statistical calculations, assuming the desired power of approximately 0.8 and the probability of type 1 error at the level of 0.05. A logistic regression analysis was performed, and the results were presented using ROC curves and the odds ratio. Receiver operating characteristic (ROC) analysis was performed to determine the predictive value of the assessed markers. The analysis was conducted with Statistica 13.0 software.

## Results

### Patient demographics

The average patient age was 66.38 ± 7.80, with 76% being males. In the AF group, the average patient age was 69.82 ± 6.42 (69% of the patients were males), and in the non-AF group, the average patient age was 65.46 ± 7.92 (76% men). The patients in the AF group were significantly older than those in the non-AF group (*P* = 0.0009) and had a lower eGFR (66.91 ± 21.90 vs. 76.47 ± 20.41). The test showed at least one comorbidity in 47 cases (43%). In the study group, the most commonly diagnosed comorbidities were type 2 diabetes in 30 patients (insulin nondependence), hypertension in 9 patients, asthma in 5 patients, and thyroid diseases in 2 patients. The characteristics of the study group are shown in Table 1.

**Table 1:** Characteristics of the whole study group and subgroups

Variable	Whole group *n* = 108		AF group (1) *n* = 23		Non-AF group (2) *n* = 85		*P*-value ^1^
	Mean (SD)	Median (IQR)	Mean (SD)	Median (IQR)	Mean (SD)	Median (IQR)	
Age [years]	–	66 (8.5)	–	71 (11.0)	–	66.0 (8.0)	*P* = 0.009
Body weight [kg]	82.08 (14.45)	–	84.91 (11.86)	–	81.3 (15.1)	–	ns
Height [m]	1.7 (0.09)	–	1.7 (0.10)	–	1.7 (0.09)	–	ns
BMI [kg/m^2^]	28.1 (4.17)	–	29.26 (4.10)	–	27.87 (4.19)	–	ns
EF [%]	54.9 (8.75)	–	52.78 (11.01)	–	55.59 (8.01)	–	ns
PVAT [mm]	7.1 (1.1)	–	7.2 (1.2)	–	7.1 (1.2)	–	ns
EuroSCORE II [pts.]	1.19 (0.83)	–	1.23 (0.9)	–	1.3 (0.79)	–	ns
Left ventricular mass [mg]	256.1 (51.1)	–	263.1 (47.1)	–	259.3 (0.82)	–	ns
eGFR [ml/min/m^2^]	74.49 (20.99)	–	66.91 (21.90)	–	76.47 (20.41)	–	*P* = 0.029

^1^- Mann-Whitney test

BMI - body mass index,

EF - Ejection Fraction,

PVAT - Perivascular Adipose Tissue,

EuroSCORE - European System for Cardiac Operative Risk Evaluation,

eGFR - Estimated Glomerular Filtration Rate,

AF- Atrial Fibrillation

SD – Standard Deviation; IQR – Interquartile Range

The obtained values of selected biochemical parameters in the examined group of patients are shown in Table 2.

**Table 2:** The values of selected biochemical parameters determined in the examined group of patients

Parameter	Whole group		AF group (1)		Non-AF group (2)		***P*** values^**1**^
	Mean (SD)	Median (IQR)	Mean (SD)	Median (IQR)	Mean (SD)	Median (IQR)	
Plasma resistin levels [ng/mL]	–	3.33 (2.71)	–	3.80 (1.43)	–	2.71(2.81)	*P* = 0.044
PVAT resistin levels [ng/g]	–	30.00 (39.27)	–	55.00 (27.00)	–	23.90 (34.57)	*P* < 0.001
Plasma IL-6 levels [pg/mL]	–	3.17 (12.27)	–	2.95 (12.37)	–	3.19 (13.20)	ns
hsCRP before surgery [mg/L]	–	2.65 (6.16)	–	1.82 (2.96)	–	2.69 (8.08)	ns
Troponin before surgery [ng/L]	–	10.25 (96.30)	–	16.90 (91.90)	–	8.40 (85.90)	ns
AspAT before surgery [IU/L]	–	28.00 (18.00)	29.95 (12.00)	–	–	29.00 (17.50)	ns
AspAT after surgery [IU/L]	–	35.00 (29.00)	–	49 (19.00)	–	33.00 (39.00)	ns
AlAT before surgery [IU/L]	–	31.00 (21.00)	29.19 (14.06)	–	–	32.00 (22.00)	ns
AlAT after surgery [IU/L]	–	29 (26.00)	–	29.00 (25.00)	–	30.00 (37.00)	ns
Bilirubin before surgery [mg/dL]	–	0.60 (0.40)	0.85 (0.31)	–	–	0.60 (0.30)	*P* = 0.012
Bilirubin after surgery [mg/dL]	–	0.80 (0.50)	0.94 (0.33)	–	–	0.75 (0.60)	ns
Protein [g/L]	7.13 (0.79)	–	7.2 (0.77)	–	7.12 (0.80)	–	ns
Glucose before surgery [mg/dL]	–	109.00 (35.00)	–	104.00 (26.00)	–	111.00 (36.00)	ns
Glucose after surgery [mg/dL]	124.62 (29.74)	–	127.40 (31.10)	–	123.78 (29.60)	–	ns
Total cholesterol [mg/dL]	–	145.00 (45.00)	146.50 (26.95)	–	–	145.00 (62.00)	ns
HDL [mg/dL]	–	38.00 (14.00)	41.92 (9.57)	–	–	37.50 (14.00)	ns
LDL [mg/dL]	–	76.50 (37.00)	77.25 (25.47)	–	–	77.00 (44.00)	ns
TG [mg/dL]	–	127.00 (64.00)	126.11 (65.86)	–	–	129.00 (67.00)	ns

^1^- Mann-Whitney test

Explanation of abbreviations: PVAT-Perivascular Adipose Tissue, AspAT- Aspartate Aminotransferase, ALAT- Alanine Aminotransferase, HDL- High-Density Lipoprotein, LDL- Low-Density Lipoprotein, TG-Triglyceride, hsCRP High-Sensitivity C-Reactive Protein WBC - White Blood Cells SD – Standard Deviation; IQR – Interquartile Range

Perivascular adipose tissue (PVAT), plasma resistin, and IL-6 values.

In the whole study group, the median resistin level in PVAT was 30.00 [ng/g]. The median PVAT resistin level was 55.00 [ng/g] in the AF group and 23.90 [ng/mL] in the non-AF group. The median resistin level in plasma was 3.33 [ng/mL] in the whole study group. The median plasma resistin level averaged 3.80 [ng/mL] in the AF group and 2.71 [ng/mL] in the non-AF group.

The median IL-6 in plasma was 3.17 [ng/mL]. In the AF group, the median IL-6 level was 2.95 ng/mL, and in the non-AF group, the median IL-6 level was 3.19 ng/mL.

Using the Mann–Whitney U test, statistically significant differences were found between the values for plasma resistin levels (*P* = 0.044) and PVAT (*P* < 0.001) in the AF group and the non-AF group. No differences appeared between the AF and non-AF groups for other parameters assessed preoperatively.

The study also assessed the correlation between resistin levels in plasma and PVAT for the whole study group and separately for the AF group and the non-AF group. Using Spearman’s rank-order correlation test, a strong positive correlation appeared between resistin levels in plasma and PVAT (r = 0.5933; *P* < 0.0001) in the whole group of patients, and a moderate correlation appeared in the AF group (r = 0.4782; *P* = 0.021) and the non-AF group (r = 0.4938; *P* < 0.0001). The analysis of the correlation between the level of resistin in PVAT and plasma, taking gender into account, showed that the correlation was present in men in the entire group (*N* = 82) (r = 0.56; *P* < 0.00001), and it did not occur in women (*N* = 26) (r = 0.37; *P* = 0.056).

### Other assessed biochemical parameters

**Fig. 2:**
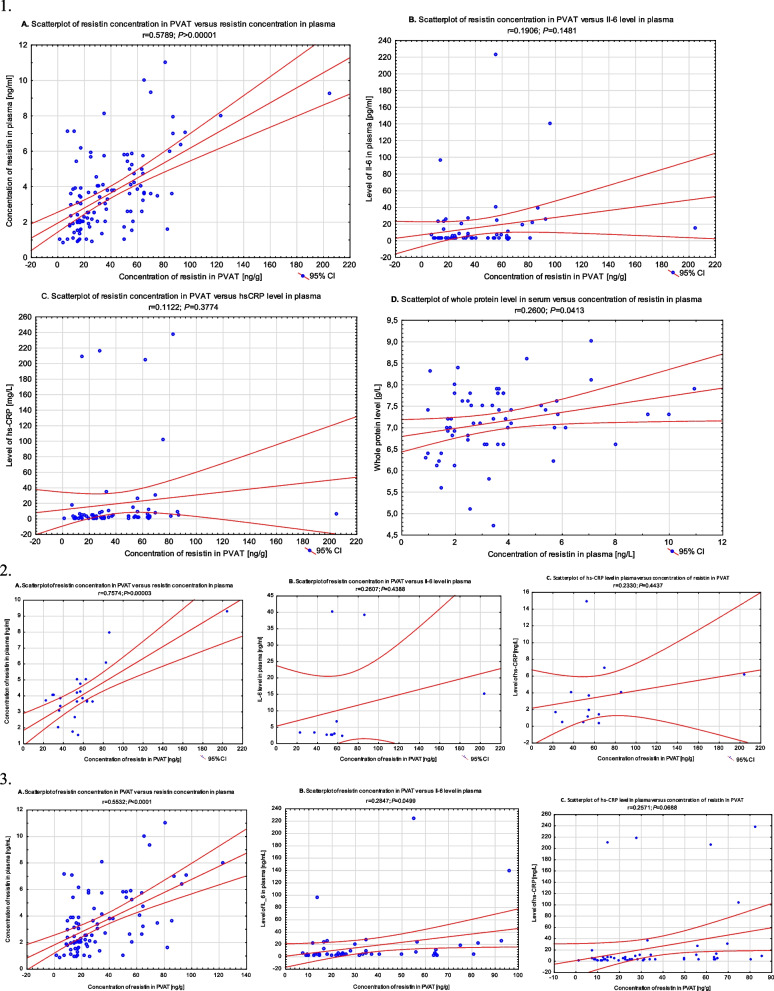
(**1**). Correlations between variables in whole group: A - Concentration of resistin in plasma and PVAT; B – level of Il-6 in plasma and concentration of resistin in PVAT; C – level of hsCRP and concentration of resistin in PVAT; D – level of whole protein in plasma and concentration of resistin in plasma. (**2**). Correlations between variables in AF group: A - Concentration of resistin in plasma and PVAT; B – level of Il-6 in plasma and concentration of resistin in PVAT; C – level of hsCRP and concentration of resistin in PVAT; (**3**). Correlations between variables in non-AF group: A - Concentration of resistin in plasma and PVAT; B – level of Il-6 in plasma and concentration of resistin in PVAT; C – level of hsCRP and concentration of resistin in PVAT

**Fig. 3:**
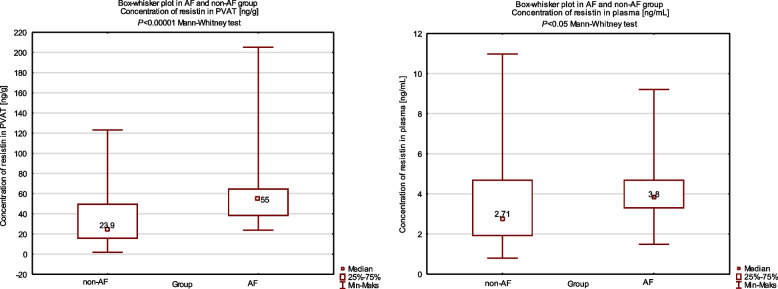
Differences in concentrations of resistin in PVAT and plasma between AF and non-AF groups (box-whisker plots)

In addition, a moderate correlation arose between the resistin level in PVAT and the hsCRP level (r = 0.3463; *P* = 0.005) in the whole study group and the non-AF group (r = 0.4448; *P* = 0.0011). There was no such correlation in the AF group (r = 0.3076; *P* = 0.306). A very weak correlation was found between the resistin level in PVAT and the IL-6 level in plasma in the non-AF group (r = 0.2957; *P* = 0.0413), while there was no such correlation in the AF group or the entire study group. A very weak negative correlation appeared between resistin levels in PVAT and total cholesterol levels (r = − 0.2847; *P* = 0.00262) in the whole study group; there was no such correlation between resistin levels in PVAT and LDL, glucose in plasma, or other cholesterol levels in the whole group or the AF and non-AF subgroups. In all patients, a moderate correlation between plasma resistin levels and total protein (r = 0.3072; *P* = 0.015) and a very weak correlation between plasma resistin levels and eGFR value (r = − 0.2725; *P* = 0.047) were present; no such correlation was found in the AF group and the non-AF group when analyzed separately. Furthermore, a strong correlation between plasma IL-6 levels and hsCRP was found in the whole study group (r = 0.5721; *P* < 0.0001) and in the non-AF group (r = 0.5623; *P* = 0.00036). A medium and low correlation was found between plasma IL-6 levels and HDL fraction levels (r = − 0.357; *P* = 0.017), and a very weak correlation appeared between hsCRP and HDL (r = − 0.2957; *P* = 0.0405) in the whole study group. The selected key correlations and differences between the AF and the non-AF groups are shown in Figs. 2 and 3.

### Logistic regression analysis

Logistic regression analysis was performed for unit variables and model fit. In the unit logistic regression analysis of variables, a statistically significant odds ratio was present for age on the day of surgery (OR = 1.09), for the level of resistin in PVAT (OR = 1.02), the eGFR value (OR = 0.9), and serum bilirubin level (OR = 6.04). The logistic regression model showed that AF was related to the patient’s age, pretreatment bilirubin levels, and PVAT resistin levels. Logistic regression analysis was also performed for demographic parameters, such as BMI and comorbidities. There was no significant influence of those variables on the analyzed regression model.

### Receiver operating characteristic (ROC) analysis

The assessment of the analyzed biomarkers’ predictive usefulness of the level of resistin in PVAT and plasma required the measurement of levels of IL-6, hsCRP, plasma troponin, LDL, CABG, body mass index (BMI), ROC curve, and AUC.

**Fig. 4:**
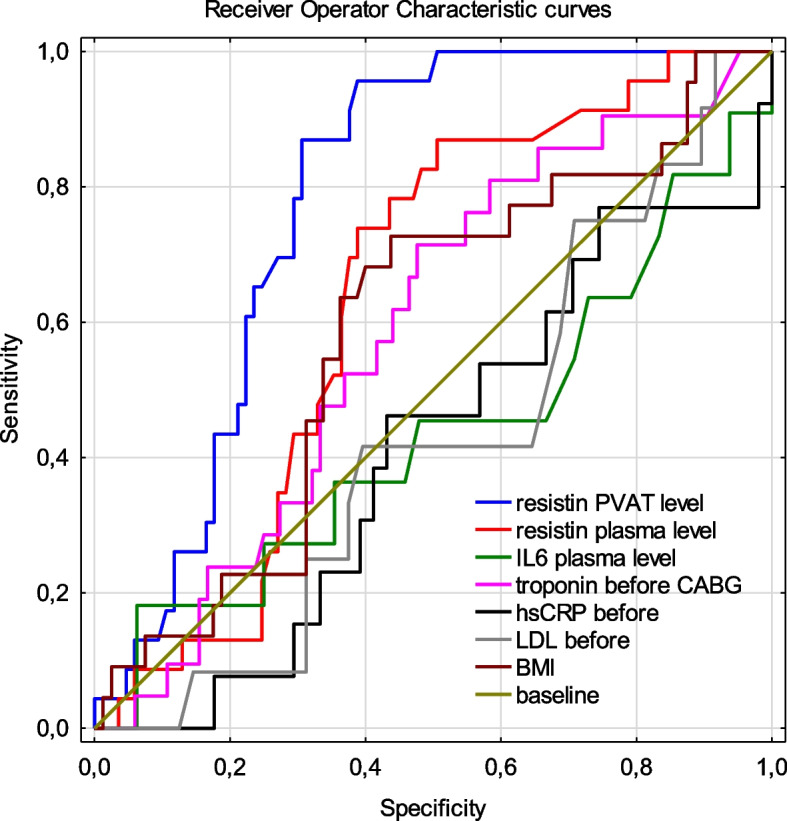
ROC curves for assessed biomarkers

The analysis of the ROC curves shows that determining the level of resistin in PVAT is the best biomarker of postoperative atrial fibrillation in patients undergoing CABG. A similarly preferred biomarker is plasma resistin levels. For both parameters, the calculated AUC is the highest; moreover, both parameters show statistical significance (*P* < 0.05) in the predictive model calculated by the Youden index. Table 3 shows the ROC analysis for the screening ability of the assessed biomarkers. Figure 4 shows ROC curves for the assessed biomarkers.

**Table 3:** ROC analysis for screening ability of the assessed biomarkers

Biomarker	AUC	*P*-value	Cutoff point	95% CI	Sensitivity	Specificity
PVAT resistin level	0.784	0.00001	30	0.7–0.868	95%	38%
Plasma resistin level	0.637	0.0156	2.6	0.526–0.748	87%	50%
Il-6	0.437	0.5462	39.08	0.23–0.643	–	–
hsCRP	0.409	0.2896	3.59	0.24–0.578	–	–
Troponin before CABG	0.585	0.1845	11.40	0.459–0.711	–	–
BMI	0.589	0.1797	28.26	0.459–0.72	–	–
LDL	0.426	0.3924	45	0.257–0.595	–	–

## Discussion

Resistin is one of the components of epicardial tissue. It is studied in terms of various correlations, both in systemic and local aspects [17]. The correlation between serum resistin levels and the patient mortality rate has yet to be fully determined. It is presumed that resistin levels in plasma do not reflect the levels in PVAT [18, 19].

According to the obtained results, the median values of PVAT resistin levels in the AF group and the non-AF group were significantly different (Spearman’s test), and they were higher in the AF group than in the non-AF group, which may indicate a significant influence of adipose tissue on the occurrence of postoperative complications.

Based on the literature on the subject, the values of the plasma resistin level in healthy persons range between 7 and 22 ng/mL, and the mean level is 15 ng/mL. In diabetic patients, the mean resistin level is estimated at 40 ng/mL [20]. For PVAT resistin levels, reference values have not been definitively developed. Previous publications compared resistin levels in PVAT sampled intraoperatively with obtained results [21].

No similar reports were found in the literature comparing resistin levels in tissue and peripheral blood. Previously published studies have mainly concentrated on determining resistin levels in peripheral blood [22]. Other researchers, in turn, assessed its presence in PVAT [19]. From a practical point of view, the publication was rather a scientific report, as it is currently impossible to analyze the composition of PVAT without its surgical collection, e.g., during cardiac surgery. Due to greater sample availability, the values of resistin levels in peripheral blood are better described in the literature, as opposed to the less frequently studied resistin levels in tissue. No publications in the literature refer to the simultaneous correlation of concentrations of potential markers of AF occurrence, such as resistin in PVAT tissue and patient plasma.

The present study also assessed the correlation between resistin and the concentration of inflammatory markers before CABG (IL-6, hsCRP, troponin). A moderate correlation appeared between PVAT resistin and hsCRP measured before surgery and plasma resistin and IL-6 before CABG. The difference between the AF and non-AF groups was not significant.

The analysis of the ROC curves showed that the highest predictive value of the compared parameters of postoperative atrial fibrillation was the level of PVAT resistin, followed by the plasma resistin level. Thus, the demonstrated correlation between these parameters confirms the usefulness of determining the plasma resistance level in patients before CABG surgery to predict the potential risk of postoperative complications.

According to Zhang et al., elevated resistin levels correlate with other inflammatory cytokines (interleukin 1 beta [IL-1 beta], IL-6, and tumor necrosis factor-alpha [TNF-alpha]). The authors experimented on an animal model and found that the development of coronary artery disease involves changes in blood flowing through the vessels as well as changes in the vessels and perivascular tissues. An explanation for this phenomenon is the penetration of proinflammatory substances circulating in plasma into the arterial wall and perivascular tissues, mainly PVAT, where the inflammatory process is likely to be localized. The same mechanism may apply to the course of atherosclerosis in humans [23].

The proinflammatory effect of resistin in plasma is linked to more frequent incidents of atrial fibrillation in the general population. The Framingham Offspring Study, in a long-term observation of a population of 2480 patients, assessed the impact of resistin concentrations on the occurrence of paroxysmal AF in the general population [24]. The correlation was present for traditional etiological factors of AF. The obtained results were similar to those in a study of postmenopausal women. Ozcan et al. and Ermakov et al. demonstrated that plasma resistin levels in patients with paroxysmal atrial fibrillation were higher than in patients without that complication [15, 22]. The role of adipocytokines as markers of inflammation was demonstrated in the example of chronic inflammatory diseases with exacerbations [25].

Similar correlations with the onset of AF were also demonstrated by Gungor et al. in a group of 40 cardiac surgery patients; however, despite the potential possibility of sampling, the study was limited to the investigation of plasma resistin levels [26]. The above papers [24, 25] present an assessment of plasma resistin concentrations and their correlation with AF. The authors do not address the potentially simultaneous relationship of plasma resistin and PVAT concentrations and possible correlations with AF.

Mazurek et al. demonstrated that the presence of inflammatory mediators in PVAT can amplify vascular inflammation, and atherosclerotic plaque instability via apoptosis (TNF-α), and neovascularization [27]. In animal studies, the adventitial application of endotoxins in vivo in pigs, monocyte chemotactic protein-1 (MCP-1), IL-1β, or oxidized LDL, induced an influx of inflammatory cells into the arterial wall, coronary vasospasm, or damage to the internal membrane, suggesting that bioactive molecules from perivascular tissues may alter arterial homeostasis [28]. The cited study provides a new method of assessing risk factors for atrial fibrillation before its onset using measurements of pro-inflammatory molecule concentrations at intravascular and extravascular levels in homogenates.

A similar paracrine effect may characterize adipocyte activity in the left atrial wall. Adipose infiltration of the atrial wall and gradual induction of inflammation lead to atrial fibrosis, which may contribute to the generation of arrhythmia [29].

According to the literature, resistin increases the production of reactive oxygen (ROS) [30]. Excessive production of ROS leads to damage to myocardial cells and the heart conduction system. According to Ren et al., ROS-induced oxidative stress links gradual atrial remodeling and paroxysmal AF [31]. Oxidative stress is generated after procedures utilizing extracorporeal circulation, such as CABG, and markers of this condition, such as myeloperoxidase (MPO), and inflammatory markers, such as IL-6, correlate with increased resistin levels after cardiac surgery. This study determined plasma resistin levels before surgery to assess the baseline concentrations and the environment in which arrhythmia may develop. Laurikka et al. found a correlation between resistin levels and IL-6 after CABG surgery, reaching its peak at 24 hours [32].

Reports suggest that plasma resistin levels are reduced by simvastatin [33]. The study by Grosso et al. showed that relatively prolonged, three-month administration of a statin with pioglitazone caused a reduction in plasma resistin levels in patients awaiting CABG surgery compared to a group of patients who did not receive such treatment. However, this therapeutic effect in circulating blood and plasma can only be achieved after prolonged treatment [34]. The presented observation suggests the advisability of determining plasma resistin levels before CABG surgery to determine the risk of postoperative AF.

In addition to qualitative studies, there are also quantitative studies of adipose tissue. Available methods make it possible to assess the thickness of adipose tissue using ultrasound, computed tomography, and MRI. For the presented group, this study was limited to the measurement of epicardial adipose tissue, which showed no significant differences in perioperative examination (Table 1).

The above observations may contribute to the deepening knowledge of AF pathogenesis and the impact of elevated resistin levels on its formation. They indicate the potential directions of therapeutic measures and interventions that may influence the composition of PVAT. It is possible to formulate a further hypothesis related to the clinical benefits, in certain patients, of reducing oxidative stress caused by extracorporeal circulation. In the future, it may be possible to design a strategy in which patients with elevated resistin levels would be recommended a revascularisation procedure utilizing *off-pump* technology instead of extracorporeal circulation. Thus, reducing the cardiovascular risk and the likelihood of AF.

It should be noted that new prophylactic drug therapies can be used for patients at risk of developing postoperative AF. They aim to mitigate the inflammatory response. Type 2 diabetic acute myocardial infarction (AMI) patients receiving sodium-glucose cotransporter 2 inhibitors (SGLT2-I) exhibited a significantly reduced inflammatory response. The inflammatory response occurring in AMI has been proposed as a potential pharmacological target [35]. In this regard, recent evidence presented in the AMI-PROTECT trial suggests that using SGLT2 in the perioperative phase of myocardial infarction may attenuate the inflammatory response and reduce the risk of arrhythmic events [36]. However, patients undergoing CABG surgery from both study groups were not taking oral SGLT2 antidiabetic drugs during the described follow-up period or before surgery. Therefore, the effect of treatment with SGLT2 inhibitors on resistin levels or IL-6 was not analyzed. An assessment of such a relationship could be included in subsequent studies.

### The strengths of the study

The strength of the paper is, in our opinion, the unique, previously unpublished study comparing plasma resistin levels with tissue resistin levels in PVAT. Such unique opportunities are only available with cardiac surgery. Once these were compared, concentrations could be related to the specific clinical problem in cardiology which is atrial fibrillation. The number of patients included in the study - 108 - is also a definite strength of the publication.

### Research limitations

1. In the future, a more detailed description of adipose tissue should be obtained with imaging tests – CT (computer tomography) and MRI (magnetic resonance imaging).
2. The present study did not assess the level of vitamin D3 in patients and its impact on their condition, which may contribute to a more in-depth observation of the causes of coronary atherosclerosis.
3. In this study, the analysis was carried out on a relatively small group of 108 patients; a more significant number would allow a more accurate assessment of the phenomenon.
4. The analysis of other adipocytokines present in PVAT would allow broadening of the spectrum of this research.

The presented results indicate the usefulness of determining plasma resistin levels, which reflect the condition of epicardial adipose tissue. Furthermore, this marker may aid in identifying patients at risk for postoperative atrial fibrillation before myocardial revascularisation.

## Supplementary Information

**Additional file 1.**

**Additional file 2.**

**Additional file 3.**

## Data Availability

Databases with research data and materials are in the possession of the co-author (MH) and can be made available if necessary.
