# Supplementary material for: Evaluation of the usefulness of determining the level of selected inflammatory biomarkers and resistin concentration in perivascular adipose tissue and plasma for predicting postoperative atrial fibrillation in patients who underwent myocardial revascularisation
Source: Lipids Health Dis. 2023 Jan 9;22:2. doi: 10.1186/s12944-022-01769-w (PMC9827643; doi:10.1186/s12944-022-01769-w)
Supplement: Supplementary file 1 — Additional file 1. [file 12944_2022_1769_MOESM1_ESM.pdf]

System Antyplagiat — Uniwersytet Medyczny im. Piastów Śląskich we Wrocławiu

Report date 9/28/2022
Edit date 9/28/2022

Accepted

Metadata

Title

Evaluation of the usefulness of determining the level of selected inflammatory biomarkers and resistin concentration in perivascular adipose tissue and plasma for predicting postoperative atrial fibrillation in patients who underwent myocardial revascularisation.

Author(s)

Maciej Rachwalik, Malgorzata Matusiewicz, Marek Jasiński, Magdalena Hurkacz

Promoter

dr Magdalena Hurkacz

Organizational unit

Uniwersytet Medyczny im. Piastów Śląskich we Wrocławiu

List of possible text manipulation attempts

In this section, you can find information regarding text modifications that may aim at temper with the analysis results. Invisible to the person evaluating the content of the document on a printout or in a file, they influence the phrases compared during text analysis (by causing intended misspellings) to conceal borrowings as well as to falsify values in the Similarity Report. It should be assessed whether the modifications are intentional or not.

Characters from another alphabet	ß	0
Spreads	A→	0
Micro spaces	·	5
White characters	ß	0
Paraphrases (SmartMarks)	a	30

Record of similarities

Please note that high coefficient values do not automatically mean plagiarism. The report must be analyzed by an authorized person.

SC1	SC2	LAD
0.00% / 5.66%	0.00% / 0.44%	0.00% / 0.00%
**25** The phrase length for the SC 2	**6183** Length in words	**39035** Length in characters

Active lists of similarities

Scroll the list and analyze especially the fragments that exceed the SC 2 (marked in bold). Use the link "Mark fragment" and see if they are short phrases scattered in the document (coincidental similarities), numerous short phrases near each other (mosaic plagiarism) or extensive fragments without indicating the source (direct plagiarism).

The 10 longest fragments

Color of the text

NO	TITLE OR SOURCE URL (DATABASE)	NUMBER OF IDENTICAL WORDS (FRAGMENTS)	
1	https://ipin.edu.pl/wp-content/uploads/2021/09/streszczenie-rozprawy-doktorskiej-m-szymanska.pdf	27	0.44 %
2	https://ipin.edu.pl/wp-content/uploads/2021/09/streszczenie-rozprawy-doktorskiej-m-szymanska.pdf	17	0.27 %
3	https://www.escardio.org/static-file/Escardio/Guidelines/Documents/ehaa612.pdf	17	0.27 %
4	The concentration of resistin in perivascular adipose tissue after CABG and postoperative atrial fibrillation<br>Małgorzata Matusiewicz,Maciej Rachwalik, Dorota Zyśko, Krzysztof Ściborski, Marta Obremska, Marek Jasiński;	16	0.26 %

5	The concentration of resistin in perivascular adipose tissue after CABG and postoperative atrial fibrillation<br>Małgorzata Matusiewicz,Maciej Rachwalik, Dorota Zyśko, Krzysztof Ściborski, Marta Obremska, Marek Jasiński;	16	0.26 %
6	https://www.spandidos-publications.com/mmr/10/2/605?text=fulltext	15	0.24 %
7	https://advances.umw.edu.pl/en/ahead-of-print/135978/	13	0.21 %
8	https://www.escardio.org/static-file/Escardio/Guidelines/Documents/ehaa612.pdf	13	0.21 %
9	https://www.mdpi.com/2076-393X/9/10/1192/htm	12	0.19 %
10	https://www.escardio.org/static-file/Escardio/Guidelines/Documents/ehaa612.pdf	12	0.19 %

from RefBooks database (2.25 %)

NO	TITLE	NUMBER OF IDENTICAL WORDS (FRAGMENTS)	
**Source: Paperity**			
1	The concentration of resistin in perivascular adipose tissue after CABG and postoperative atrial fibrillation<br>Małgorzata Matusiewicz,Maciej Rachwalik, Dorota Zyśko, Krzysztof Ściborski, Marta Obremska, Marek Jasiński;	68 (7)	1.10 %
2	Preperitoneal Blockade in the Treatment of Patients with Perforated Gastroduodenal Ulcers and Peritonitis<br>Alexei L. Charyshkin, Sergei A. Yakovlev, Vladimir P. Demin;	20 (3)	0.32 %
3	Pathophysiological role of major adipokines in Atrial Fibrillation<br>Rafaqat, Simon,Rafaqat, Saira, Rafaqat, Sana;	19 (3)	0.31 %
4	Glycaemia, arterial pressure and micro-albuminuria in Type 1 (insulin-dependent) diabetes mellitus<br>T. Gold;	11 (2)	0.18 %
5	Association of myocardial hemorrhage and persistent microvascular obstruction with circulating inflammatory biomarkers in STEMI patients<br>Pierre Croisille, Régine Cartier, Gilles Rioufol, Michel Ovize, Sylvie Espanet, Cyril Prieur, Claire Crola Da Silva, Nathan Mewton, Alexandre Paccalet, Jules Lassus,Thomas Bochaton, Eric Bonnefoy-Cudraz, François Derimay, Camille Amaz, Nathalie Dufay, Charles de Bourguignon, Hugo Bernelin;	10 (1)	0.16 %
6	Diagnostic performance of a wearing dynamic ECG recorder for atrial fibrillation screening: the HUAMI heart study<br>Li, Ruogu,Fu, Wenxia;	6 (1)	0.10 %
7	Hepcidin-25 gives an indication of the therapeutic effectiveness of tocilizumab in rheumatoid arthritis - Relationship between disease activity of rheumatoid arthritis and anemia<br>Yoshinori Kanai, Seiichiro Ando, Yoshinari Takasaki, Satoshi Suzuki, Souichiro Nakano, Ran Matsudaira,Satoshi Suzuki, Kenjiro Yamanaka;	5 (1)	0.08 %

from the Database of Legal Acts (0.00 %)

Wolters Kluwer Polska

NO	TITLE	NUMBER OF IDENTICAL WORDS (FRAGMENTS)

from the home database (0.00 %)

NO	TITLE	NUMBER OF IDENTICAL WORDS (FRAGMENTS)

from the Database Exchange Program (0.00 %)

NO	TITLE	NUMBER OF IDENTICAL WORDS (FRAGMENTS)

from the Internet (3.41 %)

NO	SOURCE URL	NUMBER OF IDENTICAL WORDS (FRAGMENTS)	
**1**	**https://ipin.edu.pl/wp-content/uploads/2021/09/streszczenie-rozprawy-doktorskiej-m-szymanska.pdf**	**50 (3)**	**0.81 %**
2	https://www.escardio.org/static-file/Escardio/Guidelines/Documents/ehaa612.pdf	42 (3)	0.68 %
3	https://www.mdpi.com/2076-393X/9/10/1192/htm	22 (2)	0.36 %
4	https://carnegieendowment.org/files/Missile_Defense_book_eng_fin2013.pdf	20 (3)	0.32 %
5	https://www.spandidos-publications.com/mmr/10/2/605?text=fulltext	15 (1)	0.24 %
6	https://advances.umw.edu.pl/en/ahead-of-print/135978/	13 (1)	0.21 %
7	https://www.tandfonline.com/doi/full/10.1080/21645515.2021.1938492	10 (1)	0.16 %
8	https://www.dovepress.com/the-role-of-stress-perception-and-coping-with-stress-and-the-quality-o-peer-reviewed-fulltext-article-PRBM	10 (1)	0.16 %
9	https://www.termedia.pl/Czynniki-demograficzne-a-jakosc-zycia-pacjentow-po-zawale-miesnia-sercowego,67,41925,1,0.html	9 (1)	0.15 %
10	https://edoc.site/cardiology-an-illustrated-textbook-jaypeepdftahir99-vrg-pdf-free.html	7 (1)	0.11 %
11	https://bmccardiovascdisord.biomedcentral.com/articles/10.1186/s12872-019-1254-5	7 (1)	0.11 %
12	https://www.ncbi.nlm.nih.gov/pmc/articles/PMC7430146/	6 (1)	0.10 %

List of accepted fragments

NO	CONTENTS	NUMBER OF IDENTICAL WORDS (FRAGMENTS)
	**https://advances.umw.edu.pl/en/ahead-of-print/13...**	**13 (0.21%)**
1	Wroclaw Medical University, Poland. 2 Department of Clinical Pharmacology, Wro...	13 (0.21%)
	**The concentration of resistin in perivascular ad...**	**68 (1.10%)**
	**https://ipin.edu.pl/wp-content/uploads/2021/09/s...**	**50 (0.81%)**
	**https://www.escardio.org/static-file/Escardio/Gu...**	**42 (0.68%)**
	**https://www.mdpi.com/2076-393X/9/10/1192/htm**	**22 (0.36%)**
	**https://carnegieendowment.org/files/Missile_Defe...**	**20 (0.32%)**
	**Preperitoneal Blockade in the Treatment of Patie...**	**20 (0.32%)**
	**Pathophysiological role of major adipokines in A...**	**19 (0.31%)**
	**https://www.spandidos-publications.com/mmr/10/2/...**	**15 (0.24%)**
	**Glycaemia, arterial pressure and micro-albuminur...**	**11 (0.18%)**
	**Association of myocardial hemorrhage and persist...**	**10 (0.16%)**

https://www.tandfonline.com/doi/full/10.1080/216...	10 (0.16%)
https://www.dovepress.com/the-role-of-stress-per...	10 (0.16%)
https://www.termedia.pl/Czynniki-demograficzne-a...	9 (0.15%)
https://edoc.site/cardiology-an-illustrated-text...	7 (0.11%)
https://bmccardiovascdisord.biomedcentral.com/ar...	7 (0.11%)
https://www.ncbi.nlm.nih.gov/pmc/articles/PMC743...	6 (0.10%)
Diagnostic performance of a wearing dynamic ECG ...	6 (0.10%)
Hepcidin-25 gives an indication of the therapeut...	5 (0.08%)
