# Supplementary material for: Evaluation of the usefulness of determining the level of selected inflammatory biomarkers and resistin concentration in perivascular adipose tissue and plasma for predicting postoperative atrial fibrillation in patients who underwent myocardial revascularisation
Source: Lipids Health Dis. 2023 Jan 9;22:2. doi: 10.1186/s12944-022-01769-w (PMC9827643; doi:10.1186/s12944-022-01769-w)
Supplement: Supplementary file 2 — Additional file 2. [file 12944_2022_1769_MOESM2_ESM.pdf]

GROY
translations

CERTYFIKAT JAKOŚCI TŁUMACZENIA

Korekta językowa tekstu pt. „Evaluation of the usefulness of determining the level of selected inflammatory biomarkers and resistin concentration in perivascular adipose tissue/epicardial adipose tissue and plasma for predicting postoperative atrial fibrillation in patients who underwent myocardial revascularisation." została wykonana przez zespół tłumaczy GROY Translations zgodnie z normą tłumaczeniową ISO PN-EN 17100:2015-06.

Korygowany tekst został sprawdzony pod względem merytorycznym, stylistycznym i terminologicznym.

Terminologia specjalistyczna użyta w tłumaczeniu została zapisana do Państwa indywidualnego słownika i będzie stosowana w przyszłych zleceniach w celu zachowania spójności i wysokiej jakości tłumaczeń. Ewentualne uwagi prosimy przesyłać na adres mailowy info@groy.pl.

TRANSLATION QUALITY CERTIFICATE

Editing of the text titled "Evaluation of the usefulness of determining the level of selected inflammatory biomarkers and resistin concentration in perivascular adipose tissue/epicardial adipose tissue and plasma for predicting postoperative atrial fibrillation in patients who underwent myocardial revascularisation." has been carried out by the GROY Translations team in accordance with the ISO PN-EN 17100:2015-06 translation standard.

The text has been verified in terms of content, style and terminology.

Specialised terminology used in the translation has been saved in your individual glossary and will be used in future assignments to ensure consistency and best translation quality. If you have any comments, please contact us at info@groy.pl.

Dziękujemy / *Thank you,*

GROY Translations

GROY group
40-224 Katowice, ul. 1 Maja 9
tel. (32) 35 21 000
NIP: 954-119-80-76
