# Supplementary material for: Evaluation of the usefulness of determining the level of selected inflammatory biomarkers and resistin concentration in perivascular adipose tissue and plasma for predicting postoperative atrial fibrillation in patients who underwent myocardial revascularisation
Source: Lipids Health Dis. 2023 Jan 9;22:2. doi: 10.1186/s12944-022-01769-w (PMC9827643; doi:10.1186/s12944-022-01769-w)
Supplement: Supplementary file 3 — Additional file 3. [file 12944_2022_1769_MOESM3_ESM.docx]

Declarations:

Approval of ethics and consent to participate:

- The study was approved by the university's bioethics committee and received the number- KB 392/2016.
- All patients agreed and filled out the appropriate forms to participate in the study.

Consent to publication:

- All authors(MR,MM,MJ,MH) agree to the review and possible publication of the above article.

Availability of data and materials:

- Databases with research data and materials are deposited with the co-author (MH) and can be made available if necessary.

Competing interests:

- In the submitted work, we do not declare competitive interests.

Financing.

- The work was written with the support of a local university subsidy with the number ST. C050.21.033

Authors' contributions:

- All authors of MM,MH,MJ,MM were involved in writing the above article. MR, MH came up with the premise of the job. MR, MH participated in the writing of all parts of the manuscript. MM performed the analysis of the material for research and helped in the section materials, methods, results. MH, MM developed statistics for publication. MJ helped substantively in writing the discussions and developing the results. MR and MH developed the graphic concept of the publication.

Thanks:

- Thanks to our graphic designer Grzegorz Kaczmarek for his help in developing illustrations for publication.
